# 13-amino derivatives of dehydrocostus lactone display greatly enhanced selective toxicity against breast cancer cells and improved binding energies to protein kinases *in silico*

**DOI:** 10.1371/journal.pone.0271389

**Published:** 2022-08-23

**Authors:** Douglas Kemboi, Moses K. Langat, Xavier Siwe-Noundou, Tendamudzimu Tshiwawa, Rui W. M. Krause, Candace Davison, Christie Jane Smit, Jo-Anne de la Mare, Vuyelwa Jacqueline Tembu

**Affiliations:** 1 Department of Chemistry, Tshwane University of Technology, Pretoria, South Africa; 2 Department of Chemistry, Rhodes University, Makhanda (Grahamstown), South Africa; 3 Royal Botanic Gardens Kew, Kew Green, Richmond, Surrey, United Kingdom; 4 Pharmaceutical Sciences Department, Sefako Makgatho Health Sciences University, Pretoria, South Africa; 5 Department of Biochemistry and Microbiology, Rhodes University, Makhanda (Grahamstown), South Africa; Vignan Pharmacy College, INDIA

## Abstract

The biological activities of dehydrocostus lactone and its analogues are suggested to be mediated by the lactone ring and *α*,*β*-methylene-*γ*-lactone. However, few studies exist on the structure-activity relationship of 13-amino derivatives of dehydrocostus latone. In this study new 13-amino derivatives of dehydrocostus lactone **DHLC** (**1–4**) were synthesized through Michael addition reactions, and were screened against three different breast cancer cell lines, namely hormone receptor positive breast cancer (MCF-7), triple-negative breast cancer (HCC70), and non-tumorigenic mammary epithelial (MCF-12A) cell lines. Dehydrocostus lactone (**DHLC**) exhibited IC_50_ values of 1.11 (selectivity index (SI) = 0.06), 24.70 (SI = 0.01) and 0.07 μM against HCC70, MCF-7 and MCF-12A cells, respectively. All the amino derivatives, except **DHLC-3** displayed low micromolar IC_50_ values (ranging from 0.07–4.24 μM) against both breast cancer cell lines, with reduced toxicity towards MCF-12A non-tumorigenic mammary epithelial cells (SI values ranging from 6.00–126.86). **DHLC-1** and D**HLC-2** demonstrated the greatest selectivity for the MCF-7 cells (with SI of 121 and 126.86 respectively) over the MCF-12A cells. This reveals that, overall, the derivatives display greatly improved selectivity for breast cancer over non-tumorigenic mammary epithelial cells, with between 100-fold and 12 000-fold higher SI values. The improved docking scores were recorded for all the 13-amino dehydrocostus lactone derivatives for the enzymes analyzed. Compounds **DHLC-4**, and **DHLC-3** recorded higher docking scores of -7.33 and -5.97 Kca/mol respectively, compared to the parent structure, dehydrocostus lactone (-5.34 Kca/mol) for protein kinase (PKC) theta (1XJD) and -6.22 and -5.88 Kca/mol, respectively for protein kinase iota (1RZR). The compounds further showed promising predicted adsorption, distribution, metabolisms and excretion (ADME) properties. Predicting the ADME properties of these derivatives is of importance in evaluating their drug-likeness, which could in turn be developed into potential drug candidates.

## Introduction

Sesquiterpene lactones are some of the most active compounds with a broad structural and functional diversity and are classified based on their skeletal structures into guaianolides, eudesmanolides, hyptocretenolides, pseudo- guaianolides, and heliangolides. However, the guaianolides are the most often reported sesquiterpene lactones with anticancer properties [1, 2].

Guaianolides were further investigated due to their interesting skeletal structures, stereochemistry, and important functional groups that are present [1–3]. The biological activities of guaianolides are suggested to be mediated by the lactone ring and an *α*,*β* or *α*,*β*,*γ*-unsaturated carbonyl structure such as *α*-methyl-γ-lactone, *α*,*β*-unsaturated cyclopentanone or some conjugate esters [2]. These functionalities are suggested to react with nucleophilic molecules such as thiol groups of the cysteine residues *via* Michael addition reactions. The Michael addition of an amine onto the enone moieties of the sesquiterpenes signifies an emerging strategy for medicinal chemists as this produces the amino-adducts that can be potential prodrugs of biologically active molecules [3]. It was further suggested that the cytotoxic agents may irreversibly alkylate critical enzymes such as protein kinase C (PKC) that control cell division. The ability to control cell division is one of the beneficial effects associated with alkylation of these compounds [2–4].

Furthermore, some structure-activity relationships of these sesquiterpenes, concerning cytotoxicity, anti-inflammatory activity, and anti-tumour activities have been investigated [5]. The findings indicated that DNA fragmentation and apoptosis-inducing activities of sesquiterpenes are mediated by an increased level of glutathione released by cells and are related to the binding between the oxo-methylene groups and thiols [5].

The *α*,*β* unsaturated carbonyl functionality in these sesquiterpenes is the common feature associated with their biological activities, which unfortunately frequently manifest itself as toxicity. However, such compounds possess poor water solubility, and the *α*-methylene-*γ*-lactone motif shows non-selective binding as a Michael acceptor with untargeted molecules. Hence, further studies on these compounds were conducted to try and overcome these problems. For instance, Woods *et al*. [3], developed an amino-prodrug synthesis strategy in which the reactive *α*,*β*-unsaturated enone was masked to enhance aqueous solubility and improve the pharmacokinetic, profile, thereby maintaining the biological activities of the parent molecule while reducing the toxicity. The conversion of the *α*,*β*-methylene-γ-lactone functional group into its amino-derivative was used as a method of protecting *α*,*β*-unsaturation from subsequent hydrogenation [3].

**Fig 1:**
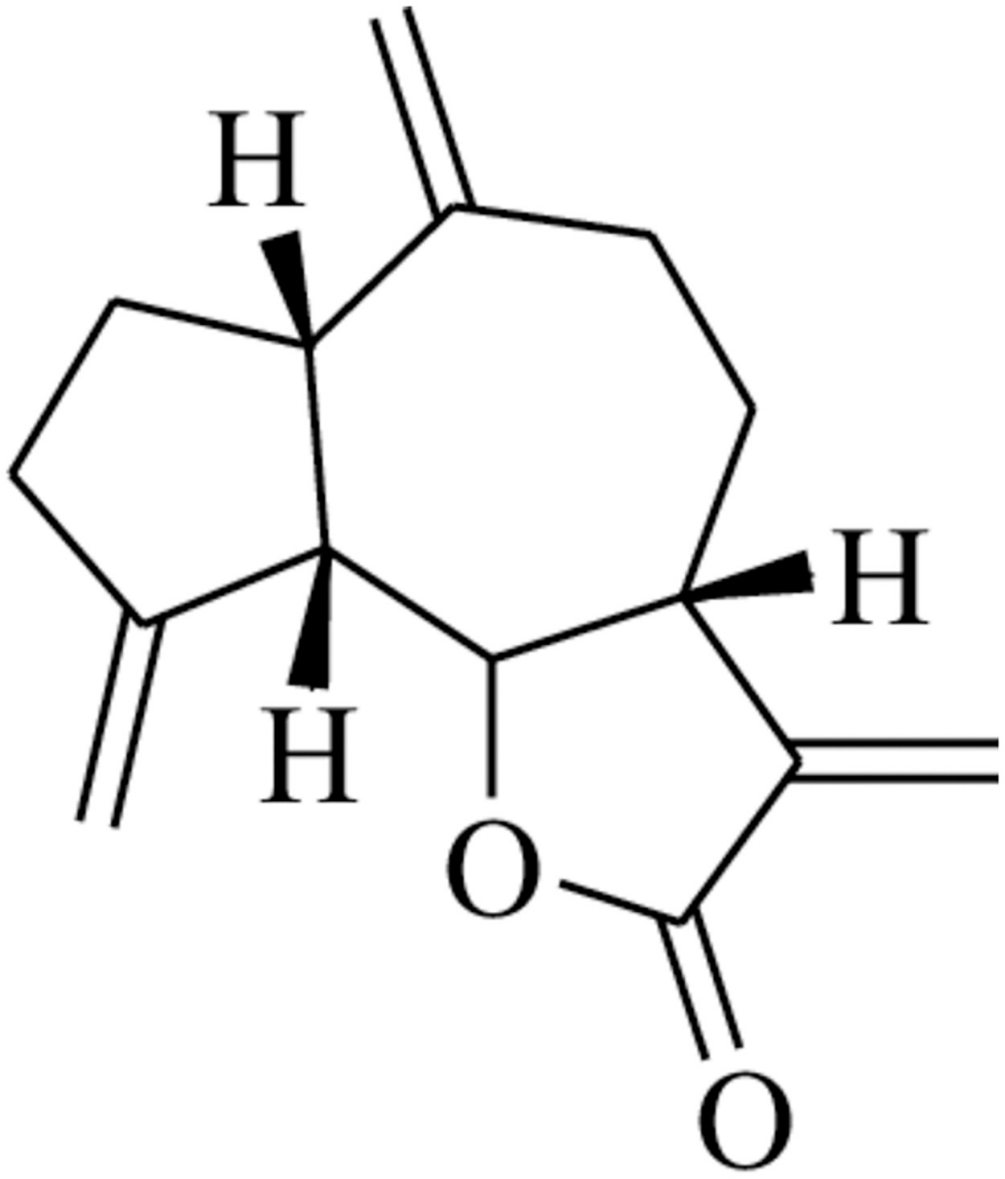
Structure of dehydrocostus lactone (DHLC).

Dehydrocostus lactone {(**DHLC**) Fig 1} a natural sesquiterpene lactone present in many medicinal plants such as *Saussurea lappa* and *Laurus nobilis* is of interest to researchers thanks to its promising biological activities, such as antiinflammation [6], and anticancer [7]. **DHLC** has been found to have a good pharmacological effects against a wide range of cancer types *in vitro*. These activities were attributed to the *α*-methylene-γ-lactone, which is the major functional group. Studies have, however, shown that **DHLC** exhibited anti-cancer effects *via* the suppressor of cytokinase signaling protein (SOCS) mediated cell cycle arrest and apoptosis [7]. Due to the double bond of α-methylene-γ-butyrolactone, **DHLC** can be used as an electrophilic group targeting the nucleophilic group present in the active site in the organism, so as to change the structure of these active sites and hence present different biological effects. Nevertheless, the study of **DHLC**’s potential cytotoxic activities on kidney and ovarian epithelial cells revealed its negative effects on normal healthy cells [8]. Moreover, **DHLC** was reported to induce apoptosis in keratinocytes that possess normal tissue origin [9].

However, no studies on the anticancer activities against breast cancer cells of dehydrocostus lactone amino derivatives possessing *α*, *β*-methylene-γ-lactone moiety have been reported. In this study, efforts were made to investigate the anticancer activities of 13-amino derivatives of dehydrocostus lactone against hormone receptor positive breast cancer cells and non-tumorigenic cancer cells as well as studying the *in silico* molecular docking properties using protein kinase C enzymes {(PKC theta (1XJD) and 1ZRZ)}.

## Materials and methods

### General preparation of amines (strategy one)

To a 200 mL round bottom flask containing 60 mL methanol, a mixture of **DHLC** (30 mg), and dimethylamine (4 mL) was added at once at room temperature. The mixture was then heated to reflux for 24 hours. The resultant mixture was then poured into water and was extracted three times with 50 mL of ethyl acetate, the organic extracts were combined and the solvent evaporated in *vacuo*. The concentrated crude product was further purified by column chromatography using a step gradient solvent system of ethyl acetate/hexane and the resultant product was allowed to dry in the fume hood [10, 11].

### Preparation of amides derivatives (strategy two)

To a 200 mL round bottom flask, was added **DHLC** (30 mg) and 0.5 mL of amine (diethylamine, trimethylamine, ethylenediamine and *N*,*N*-dimethylacetamide), each dissolved in absolute ethanol (60 mL). The reaction mixtures were then heated to reflux for 6 hours and stirred at room temperature overnight for 12 hours, after which excess ethanol was removed *in vacuo*. The crude products were further purified by column chromatography using a step gradient solvent system of absolute ethanol and hexane of varying ratios [10, 11].

### Resazurin assay

Cytotoxicity and the IC_50_ of the synthetic compounds were determined using the resazurin assay, [12, 13]. The cancer cells MCF-7 hormone receptor positive breast cancer [estrogen receptor (ER)^+^, progesterone receptor (PR)^+^, human epidermal growth factor-2 (HER-2)^-;^ ATCC: HTB-22), HCC70 triple negative breast cancer (TNBC, ER^-^, PR^-^, HER-2^-^; ATCC: CRL-2315) and a non-tumorigenic breast epithelial cell line MCF-12A (ATCC: CRL-10782), were seeded at a density of 5000 cells/well in a 96 well plate and were allowed to adhere overnight at 37°C in a 9% CO_2_ incubator. The cells were then treated with the synthetic compounds at a concentration range from 15.63–500.00 μM or with a vehicle control [0.2% (v/v) DMSO] for 96 hours at 37°C in a 9% CO_2_ incubator. Thereafter, 0.54 nM of resazurin solution was added and the cells were incubated for 2–4 hours at 37°C in a 9% CO_2_ incubator. The fluorescence was then measured on a Spectramax spectrophotometer with excitation and emission wavelength set at 560 and 590 nm respectively. The experiment was done in technical triplicate and the data were analyzed using GraphPad Prism Inc, (USA) with half-maximal inhibitory concentration (IC_50_ values) determined by non-linear regression. Selectivity index values for the compounds were calculated as follows: (IC_50_ of compound against MCF12A cells)÷(IC_50_ of compound against breast cancer cells) where a SI > 1 is indicative of selective toxicity towards cancer cells vs non-cancerous cells.


$$Selectivity\;index=\frac{half-maximal\;inhibitory\;concentration\;against\;MCF12A\;cells}{half-maximal\;inhibitory\;concentration\;against\;breast\;cancer\;cells}$$

### *In silico* molecular docking studies

In this study, the binding strength, binding poses, and protein-ligand interactions were investigated. *In silico* docking was done on protein kinases (1ZRZ) and the PKC theta (1XJD) transferase enzymes. The enzymes were chosen based on promising scientific evidence showing that these isoenzymes can be activated by sesquiterpenes lactones [3, 4].

The three-dimensional protein crystal structures were downloaded from the RCSB Protein Data Bank [14]. The enzymes occur as dimeric structures, hence, Chain A was selected for the computational studies. Discovery Studio Visualizer [15] was used in determining the center of mass of crystal ligand in the active site of A Chain. Other components of the crystal structure like Chain B of the dimer, crystal structure ligand, and crystal structure water, were also removed using Discovery Studio Visualizer [15]. Chain A of the receptor was prepared for docking using the protein preparation wizard as implemented in Maestro. The receptor grid generations were achieved using “Glide Grid Generation” module and the active site was selected with a radius of 20 Å around the crystal ligand. The ligand structures utilized for docking were the PKC theta (1XJD) and 1ZRZ and they were prepared for docking using the LigPrep module (Schrodinger, LLC, NY, USA, 2009). The OPLS3e force field was used as implemented in LigPrep for the energy minimization of the ligands to generate low-energy ligand isomers, the addition of hydrogens, charges, and generation of possible ionization states and tautomers. Protein-ligand docking was then performed in Maestro with glide using standard precision (SP) with flexible ligands. The results obtained were visualized and analyzed in Maestro.

### General experimental procedure

Column chromatography was carried out on polyamide columns (Germany GmbH) over silica gel (Kieselgel 60 Å GF_254_, pore size 35–75 μm particle size, Merck, Germany), while Thin Layer Chromatography (TLC) was performed on Kieselgel 60 F_254_; Merck) to a thickness of 0.25 mm. Active spots on UV active silica gel were visualized under ultraviolet (UV) light (245 and 336 nm). Dehydrocostus lactone (**DHLC**), synthetic reagents, and all solvents (hexane, ethyl acetate, dichloromethane and absolute ethanol) used for column chromatography were purchased from Merck and Sigma, South Africa and were used as received.

The nuclear magnetic resonance (NMR) spectra for 1D (^1^H and ^13^C) and 2D (COSY, HSQC, HMBC and NOESY) of all compounds were obtained on Bruker Avance III HD NMR spectrometer at 400 MHz at Rhodes University, Chemistry department, South Africa. Approximately 5.0–15.0 mg of each pure compound (**DHLC-1**-**4**) was dissolved in deuterated chloroform solvent (CDCl_3_). The NMR spectra are recorded at 25°C while the chemical shifts (δ) were expressed in parts per million (*ppm*) and are referenced to the internal solvent shift in ^1^H and ^13^C (δ 0.0 *ppm*) of tetramethylsilane (TMS). Coupling constants are reported in hertz (Hz) and multiplicities are reported as singlet (*s*), doublet (*d*), doublet of doublet (*dd*), triplet (*t*), and multiplet (*m*). Infra-Red (IR) spectra were measured using Perkin-Elmer spectrometer, version 10.54. The spectrum was then identified and analyzed by OMNIC spectra software in the spectrometer system. The infrared absorptions were reported in wavenumbers (cm^-1^).

Specific optical rotation [*α*]_D_ was performed on Jasco P-2000 polarimeter. The angle of rotation *α* of polarized light was recorded at 200 ± 0.50 in chloroform solution and was expressed in degrees (°) of the plane of polarization at the wavelength of 546.3 nm of the D-line of sodium.

The high-resolution electron spray ionization mass spectroscopic (HR-ESI-MS) analysis of the synthetic compounds was analyzed on a Bruker Daltonics Compact QTOF mass spectrometer in positive mode using an electrospray ionization probe. The spectrometer was coupled to a thermal scientific ultimate 3000 Dionex UHPLC system which consisted of an RS Auto Sampler WPS-3000, Pump HPG-3400 RS and detector DAD-3000 RS. Processed spectra of all reported compounds are shown in supplementary materials A1-A44 in S1 File.

The HCC70 (ATCC-CRL-2315) basal triple-negative breast cancer (TNBC) cell lines and MCF-7 luminal A cancer cell line (ATCC-HTB-22) were purchased from the American Type Culture Collection (ATCC, USA). The MCF-12A breast epithelial cell line (ATCC-CRL-10782) was a gift from Dr Anna-Mart Engelbrecht (Department of Physiology, Stellenbosch University, South Africa).

### Spectral data

**Guaia-4(15),10(14)-dien-12-oic acid, 13-(dimethylamino)-6-hydroxy-γ-lactone (DHLC-1)**. ^1^H NMR (400 MHz, CDCl_3_, δ *ppm*): 2.72 (1H, *m*, 1-H), 1.98 (1H, *m*, 2-H), 3.86 (1H, *t*, *J* = 2.1, 3-H), 2.83 (1H, *m*, 5-H), 2.75 (1H, *t*, *J* = 6.4, 6-H), 2.63 (1H, *m*, 7-H), 2.39 (2H, *m*, 8-H), 2.51 (2H, *m*, 9-H), 2.73 (1H, *t*, *J* = 1.9, 11-H), 2.64 (2H, *d*, *J* = 1.2, 13-H), 4.79 (1H, *brs*, 14-H), 4.96 (1H, *brs*, 15-H), 2.17 (3H. *s*, 16-H), 2.17 (1H, *s*, 17-H). ^13^C NMR (100.6 MHz, CDCl_3_, δ *ppm*): 47.1 (C-1), 32.6 (C-2), 30.2 (C-3), 151.9 (C-4), 52.0 (C-5), 85.4 (C-6), 45.5 (C-7), 32.9 (C-8), 37.8 (C-9), 150.6 (C-10), 46.9 (C-11), 177.7 (C-12), 58.8 (C-13), 109.2 (C-14), 111.7 (C-15), dimethylamine (45.9, 45.9) (S1 Table in S1 File).

(*3a*S*,6a*R*,9a*R*,9bS*)-3-[(diethylamino)methyl]decahydro-6,9-bis(methylene)azuleno[4,5-b]furan-2(3*H*)-one (DHLC-2). ^1^H NMR (400 MHz, CDCl_3_, δ *ppm*): 2.78 (1H, *m*, 1-H), 2.40 (2H, *m*, 2-H), 3.82 (1H, *m*, 3-H), 2.51 (1H, *m*, 5-H), 2.71 (1H, *t*, *m*, 6-H), 2.39 (1H, *m*, 7-H), 3.87 (2H, *m*, 8-H), 2.15 (2H, *m*, 9-H), 2.77 (1H, *m*, 11-H), 2.48 (2H, *m*, 13-H), 4.79 (1H, *brs*, 14-H), 5.12 (1H, *brs*, 15-H), 2.41 (3H. *m*, 16-H), 2.34 (1H, *m*, 17-H), 0.92 (3H, *t*, *J* = 19.4, H-18, 19). ^13^C NMR (100.6 MHz, CDCl_3_, δ *ppm*): 47.1 (C-1), 32.6 (C-2), 30.2 (C-3), 151.8 (C-4), 52.1 (C-5), 85.4 (C-6), 45.9 (C-7), 33.0 (C-8), 37.7 (C-9), 150.2 (C-10), 45.9 (C-11), 177.9 (C-12), 52.9 (C-13), 109.1 (C-14), 111.6 (C-15), diethylamine (47.1, 11.7, 11.7) (S1 Table in S1 File).

**Guaia-4(15),10(14)-dien-12-oic acid, 13-(ethylamino)-6-hydroxy-γ-lactone (DHLC-3)**. ^1^H NMR (400 MHz, CDCl_3_, δ *ppm*): 2.91 (1H, *m*, 1-H), 1.39 (2H, *m*, 2-H), 2.54 (1H, *t*, *J* = 2.1, 3-H), 2.85 (1H, *m*, 5-H), 3.98 (1H, *t*, *J* = 6.0, 6-H), 2.25 (1H, *m*, 7-H), 2.16 (2H, *m*, 8-H), 2.51 (2H, *m*, 9-H), 2.73 (1H, *t*, *J* = 1.9, 11-H), 2.91 (1H, *d*, *J* = 1.2, 13-H), 5.07 (1H, *brs*, 14-H), 4.90 (1H, *brs*, 15-H), 1.37 (2H. *m*, 16-H), 1.16 (3H, *t*, *J = 8*, 17-H). ^13^C NMR (100.6 MHz, CDCl_3_, δ *ppm*): 47.8 (C-1), 32.5 (C-2), 30.2 (C-3), 151.8 (C-4), 51.9 (C-5), 85.8 (C-6), 45.4 (C-7), 32.7 (C-8), 37.6 (C-9), 149.7 (C-10), 47.8 (C-11), 178.0 (C-12), 47.8 (C-13), 109.2 (C-14), 111.9 (C-15), ethylamine (44.3, 14.9) (S1 Table in S1 File).

**13-ethylenediamine dehydrocostus lactone (DHLC-4)**. ^1^H NMR (400 MHz, CDCl_3_, δ *ppm*): 2.37 (1H, *m*, 1-H), 2.54 (2H, *m*, 2-H), 1.97 (1H, *t*, *J* = 2.1, 3-H), 2.83 (1H, *m*, 5-H), 3.96 (1H, *t*, *J* = 6.0, 6-H), 2.42 (1H, *m*, 7-H), 2.50 (2H, *m*, 8-H), 2.07 (2H, *m*, 9-H), 2.73 (1H, *t*, *J* = 1.9, 11-H), 2.88 (1H, *d*, *J* = 1.2, 13-H), 5.06 (1H, *brs*, 14-H), 4.89 (1H, *brs*, 15-H), 2.71 (1H. *m*, 16-H), 2.82 (1H, *m*, 17-H). ^13^C NMR (100.6 MHz, CDCl_3_, δ *ppm*): 47.5 (C-1), 32.5 (C-2), 30.2 (C-3), 151.9 (C-4), 51.9 (C-5), 85.4 (C-6), 45.0 (C-7), 32.7 (C-8), 37.7 (C-9), 150.7 (C-10), 49.4 (C-11), 177.8 (C-12), 47.7 (C-13), 109.2 (C-14), 112.0 (C-15), ethylenediamine (52.7, 41.6) (S1 Table in S1 File).

## Results and discussion

### Synthesis of 13-amino derivatives of dehydrocostus lactone (DHLC)

The desired decrease in toxicity and increase in activity of **DHLC** was achieved through amination (Scheme 1) of the double bond.

Scheme 1. Amination of the double bond.



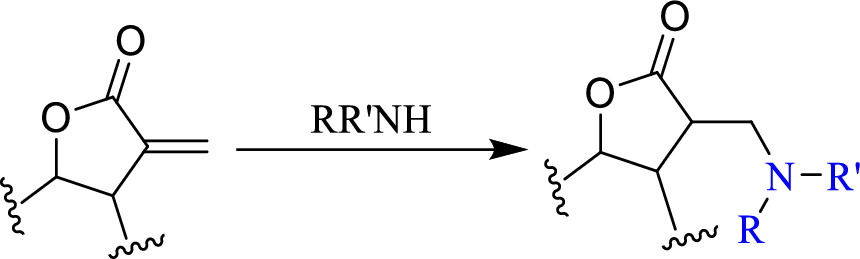



The amination of the C-13 double bond of **DHLC** was achieved using primary, secondary, and tertiary amines. Nitrogen atoms from different amines; dimethylamine, diethylamine, ethylamine, triethylamine, ethylenediamine, diisopropylamine, benzylamine, and 2-methoxybenzylamine were introduced at the C-13 double bond of **DHLC** to yield respective amino derivatives as in Scheme 2, Table 1 and Fig 4. Primary amines derivatives and secondary amine derivatives were obtained in good yields of between 50–80%. Tertiary amines (triethylamine) did not react with **DHLC** even after refluxing the reaction mixture for a further 24 hrs as the starting materials were recovered. Previous studies reported that cyclic and secondary amines possess more predictable conformation and can be used to construct stereoselective amine derivatives [16, 17] which were consistent with our findings.

**Table 1:** Physical properties of 13-amino dehydrocostuslactone derivatives.

Entry	Amine name	Time (hr)	Yield (%)	Optical rotation	IR (cm^-1^)
**DHLC-1**	Dimethylamine	12	65	-15.54	3378 (N-H), 2934 (C-H), 1768 (C = H),
**DHLC-2**	Diethylamine	12	62	-107.46	3331 (N-H), 2969 (C-H), 16658 (C = H)
**DHLC-3**	Ethylamine	12	75	-40.60	3374 (N-H), 2922 (C-H), 1772 (C = H),
**DHLC-4**	Ethylenediamine	24	54	-43.15	3240 (N-H), 2899 (C-H), 1466 (C = H),

Scheme 2. Amination of C-13 double bond of dehydrocostus lactone.



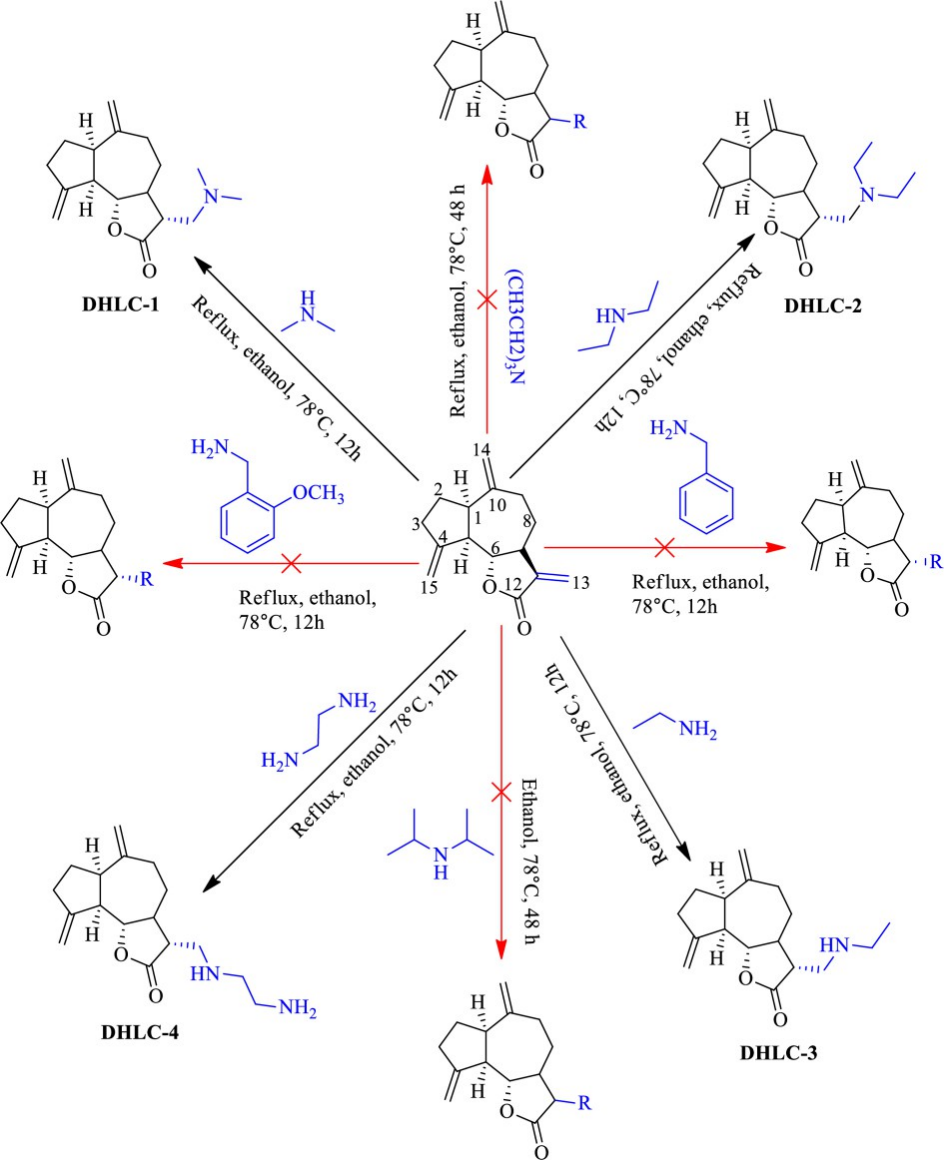



### Characterization of synthesized compounds

**Fig 2:**
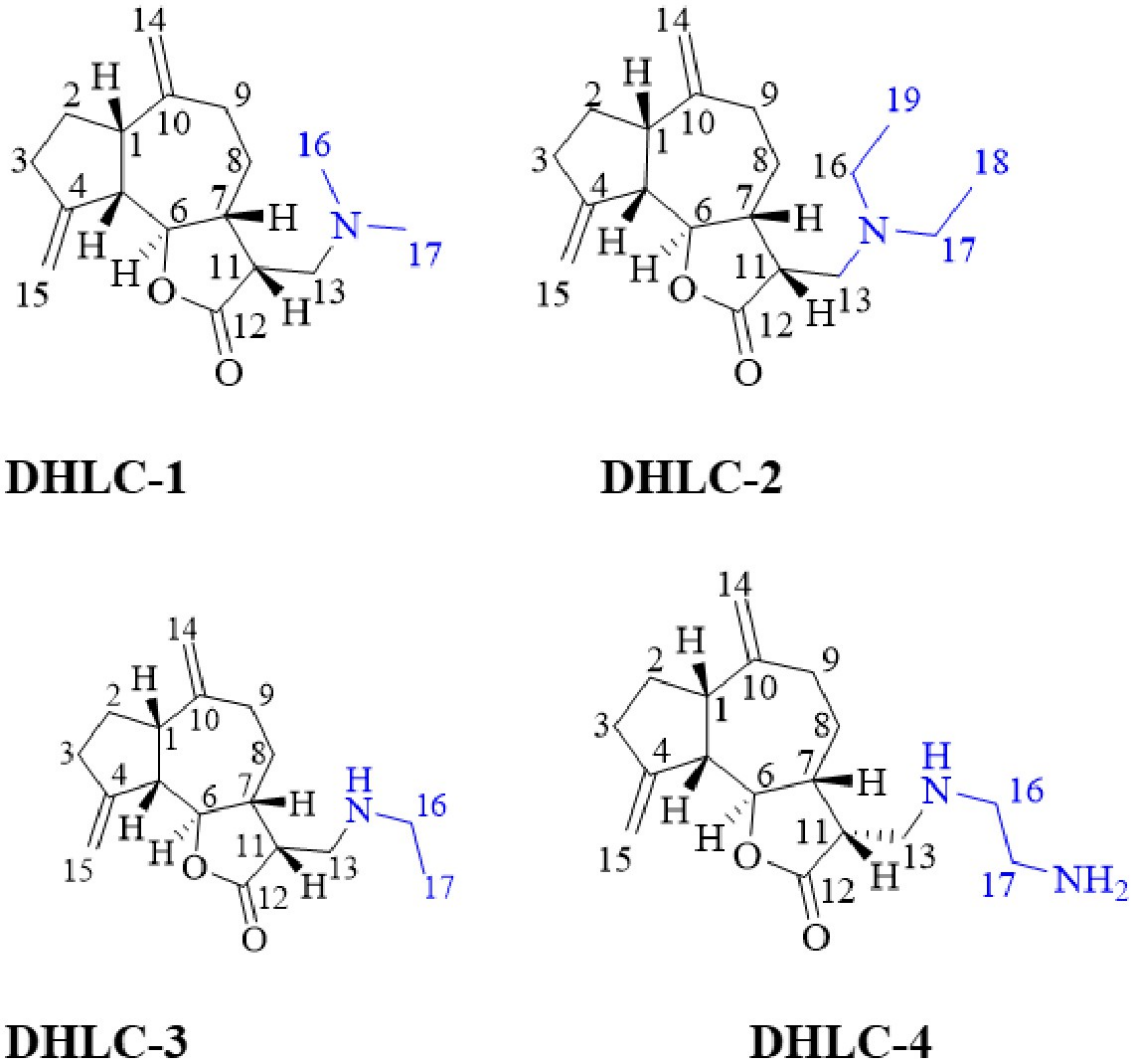
Structures of synthesized derivatives of dehydrocostus lactone.

The amination of the C-13 double bond of **DHLC** resulted in successful synthesis of guaia-4(15),10(14)-dien-12-oic acid, 13-(dimethylamino)-6-hydroxy-γ-lactone (**DHLC-1)**, (*3a*S*,6a*R*,9a*R*,9bS*)-3-[(diethylamino)methyl]decahydro-6,9-bis(methylene)azuleno[4,5-b]furan-2(3*H*)-one (**DHLC-2)**, guaia-4(15),10(14)-dien-12-oic acid, 13-(ethylamino)-6-hydroxy-γ-lactone (**DHLC-3)**, and 13-ethylenediamine dehydrocostus lactone (**DHLC-4)**. The structures of the synthesized compounds are provided in Fig 2.

**DHLC-1** was obtained as a colourless oil with Rf value of 0.53 (8:1; EtOH: Hexane), 45.1mg, 65% yield with its specific optical rotation recorded at[*α*]_D_^20^–15.54 {CHCl_3_, Conc = 31.00 (w/v%}]. The IR spectrum showed weak N-H stretches at 3215 cm^-1^. The absorptions bands at 2916 and 2854 cm^-1^ were attributed to C-H (alky) stretches and 1460 cm^-1^ due to C = C stretches (Appendix A16 in S1 File). The HR-ESI-MS displayed a pseudomolecular ion [M+H]^+^ at *m/z* 276.1996 which indicated a molecular formula of C_17_H_25_NO_2_ (Appendix A17 in S1 File). The NMR data of compound **DHLC-1** are provided in Appendices A9-A15 in S1 File.

Analysis of the ^1^H NMR spectrum of compound **DHLC-1** exhibited singlet at δ_H_ = 2.35 and integrating six protons. This was attributed to the dimethyl groups of the dimethylamine. The absence of olefinic proton signals at δ_H_ 5.52 and δ_H_ 6.52 of dehydrocostus lactone confirmed the introduction of an amine group at C-13 in dehydrocostus lactone. This was further confirmed by the presence of a methyl carbon resonance at δ_C_ 45.9, attributed to methyl groups of the dimethylamine. In addition, the absence of olefinic carbon signals at δ_C_ 120.9 and 139.1 confirmed the formation of 13-dimethylamine dehydrocostus lactone as illustrated in S1 Table in S1 File.

Compound **DHLC-2** was obtained as a colourless oil with Rf of 0.54 (8:1; EtOH/Hexane), 42.2 mg, 62% yield with its specific optical rotation recorded as [*α*]_D_^20^–107.46 {CHCl_3_, Conc = 4.10 (w/v%)}. The IR spectrum showed N-H absorption bands at 3300 cm^-1^. The absorptions bands at 2913 and 2858 cm^-1^ were attributed to C-H (alky) stretches while bands at 1636 and 1460 cm^-1^ were due to C = C stretches (Appendix A26 in S1 File). The HR-ESI-MS exhibited a pseudomolecular ion [M+Na]^+^ at *m/z* 326.0898 which indicated a molecular formula of C_19_H_29_NO_2_ (Appendix A25 in S1 File). The NMR data of compound **DHLC-2** are provided in Appendices A18-A24 in S1 File.

Analysis of the ^1^H NMR spectrum of the compound exhibited a triplet signal at δ_H_ 0.93 integrating to six protons. This was attributed to the two methyl groups of the diethylamine moiety. Multiplet proton peaks at δ_H_ 2.49 were attributed to methylene protons H-16 and H-17 of the diethylamine. The absence of olefinic proton peaks at δ_H_ 5.52 and δ_H_ 6.52 of dehydrocostus lactone confirmed the introduction of an amine group at C-13 in dehydrocostus lactone. This was further confirmed by the presence of a methyl carbon peak at δ_C_ 11.7 ppm, attributed to two methyl groups of the diethylamine. In addition, a methylene carbon peak at δ_C_ 47.2 was attributed to the methylene carbons of diethylamine and this helped to confirm the successful addition of diethylamine at C-13. The carbon values compared well with previously synthesized diethylamine of dehydrocostus lactone [18] as in S1 Table in S1 File.

Compound **DHLC-3** was obtained as a colourless oil with Rf of 0.43 (C_6_H_14_: CH_2_Cl_2_ (8:2)), 55.1mg with its optical specific rotation recorded as [*α*]_D_^20^–40.60 {CHCl_3_, Conc = 2.50 (w/v%)}. The IR spectrum showed N-H stretches at 3275 cm^-1^. The absorptions bands at 2922 and 2847 cm^-1^ were attributed to C-H (alky) stretches while 1642 cm^-1^ was due to C = C stretches (Appendix A35 in S1 File). The HR-ESI-MS exhibited a molecular ion [M+H]^+^ at *m/z* 276.1927 which indicated a molecular formula C_17_H_25_NO_2_ (Appendix A34 in S1 File). The NMR data of compound **DHLC-3** are provided in Appendices A27-A33 in S1 File.

Analysis of the ^1^H NMR spectrum exhibited a triplet signal at δ_H_ 1.16 ppm. This was attributed to the methyl group of the ethylamine. The absence of olefinic proton signals at δ_H_ 5.52 and δ_H_ 6.52 of dehydrocostus lactone and the presence of signals at δ_H_ 2.91 (H-13) and δ_H_ 2.72 (H-16) confirmed the introduction of an amine group at C-13 in dehydrocostus lactone. This was further confirmed by the presence of a methyl carbon signal at δ_C_ 14.9 (C-17) and δ_C_ 44.3 (C-13), attributed ethyl group of the ethylamine. The absence of olefinic carbon signal at δ_C_ 120.9 and 139.1 confirmed the successful addition of ethylamine at this position as in S1 Table in S1 File.

Compound **DHLC-4** was obtained as a colourless oil with Rf of 0.51 (C_6_H_14_: CH_2_Cl_2_), 35.7 mg, 54% yield with its optical specific rotation recorded as [*α*]_D_^20^–43.15 {CHCl_3_, Conc = 8.50 (w/v%)}. The IR spectrum showed N-H stretches at 3240 cm^-1^. The absorptions bands at 2898 cm^-1^ were attributed to C-H (alky) stretches while 1 due to C = C stretches (Appendix A44 in S1 File). The HR-ESI-MS displayed a pseudomolecular ion [M+H]^+^ at *m/z* 291.9881 which indicated a molecular formula of C_17_H_26_N_2_O_2_ (Appendix A43 in S1 File). The NMR data of compound **DHLC-4** are provided in Appendices A36-A42 in S1 File.

Analysis of the proton spectrum exhibited signals at δ_H_ 2.71 and δ_H_ 2.82 due to the methylene protons of C_2_H_4_(NH_2_)_2_. The absence of olefinic proton signals at δ_H_ 5.52 and δ_H_ 6.52 of dehydrocostus lactone and the presence of signals at δ_H_ 2.71 (*m*, H-16) and δ_H_ 2.82 (*m*, H-17) confirmed the presence of an ethylenediamine group at C-13 in dehydrocostus lactone (S1 Table in S1 File). This was further confirmed by the presence of carbon signals at δ_C_ 47.7 (C-13), δ_C_ 41.6 (C-17), and δ_C_ 52.7 (C-16), which were attributed to the ethylenediamine substituent at C-13. In addition, the absence of olefinic carbon signals at δ_C_ 120.9 and 139.1 confirmed the addition of C_2_H_4_(NH_2_)_2_ at C-13.

### Cytotoxic activities of 13-amino derivatives of dehydrocostus lactone

Dehydrocostus lactone (**DHLC**) and all synthesized amino derivatives were evaluated *in vitro* for their cytotoxicity against breast cancer cells including the HCC70 and MCF-7 cell lines, and non-tumorigenic mammary epithelial (MCF-12A) cells using camptothecin as a positive control. The cytotoxicity data for all the synthetic derivatives are summarized in Table 2. It is evident from the findings that the parent molecule, dehydrocostus lactone (**DHLC**) displayed cytotoxic activities against HCC70, MCF-7, and MCF-12A with IC_50_ values of 1.11 ± 1.31, 24.70 ± 1.25, and 0.07 ± 0.07 μM respectively. Compounds **DHLC-1** and **DHLC-2** exhibited cytotoxic activities against HCC70, MCF-7 and MCF-12A with IC_50_ values of 0.64 ± 1.47, 0.07 ± 0.07 and 8.47 ± 1.24, respectively in the case of **DHLC**-1 and 1.48 ± 1.49, 0.07 ± 1.31 and 8.88 ± 1.10 μM, respectively, in the case of **DHLC-2**, as illustrated in Table 2. With the exception of **DHLC-3**, all of the derivatives displayed low micromolar IC_50_ values (ranging from 0.07–4.24 μM) against HCC70, and MCF-7 breast cancer cell lines, with reduced toxicity towards non-tumorigenic mammary epithelial (MCF-12A) cells. The derivatives **DHLC-1**, **DHLC-2**, **DHLC-3** and **DHLC**-4 demonstrated selectivity for the HCC70 [with selectivity index (SI) values of 13.23, 6.00 57.91 and 54.50, respectively] and MCF-7 breast cancer cells (with SI values of 121, 126.86, 8.33 and 34.83, respectively) over the MCF-12A non-cancerous cells. The parent compound displayed SI values of 0.06 and 0.01 for HCC70 and MCF-7 cells, respectively. This reveals that, overall, the amino derivatives of dehydrocostus lactone display greatly improved selectivity for breast cancers (HCC70, and MCF-7) over non-tumorigenic mammary epithelial cells (MCF-12A) compared to the parent compound, ranging from 100- to over 12 000-fold higher SI values.

**Table 2:** Anticancer activities of 13-amino derivatives compounds against HCC70, MCF-7, and MCF12A cell lines.

Compound Name	HCC70		MCF-7		MCF-12A	
	IC_50_ (μM) ± SD	R^2^	IC_50_ (μM) ± SD	R^2^	IC_50_ (μM) ± SD	R^2^
**DHLC-1**	0.64 ± 1.47	0.9990	0.07 ± 0.07	0.9021	8.47 ± 1.42	0.845
	SI = 13.23		SI = 121			0
**DHLC-2**	1.48 ± 1.49	0.9738	0.07 ± 1.31	0.9881	8.88 ± 1.10	0.914
	SI = 6.00		SI = 126.86			0
**DHLC-3**	3.25 ± 1.36	0.9738	222. 60 ± 1.01	0.8006	188.20 ± 1.35	0.708
	SI = 57.91		SI = 8.33			0
**DHLC-4**	2.71 ± 1.30	0.9977	4.24 ± 1.29	0.8964	147.70 ± 1.28	0.856
	SI = 54.50		SI = 34.83			3
**DHLC**	1.11 ± 1.31	0.9993	24.70 ± 1.25	0.9027	0.07 ± 0.07	0.930
	SI = 0.06		SI = 0.01			3
**Camptothecin**	83.17 ± 1.08	0.9933	103.8 ± 9.92	0.9920	104.2 ± 1.04	0.9938

Key: SI = selectivity index

The type of amine group used produced 13-amino derivatives with varying cytotoxicity activities. For instance, 13-ethylamine derivative (**DHLC-3**) exhibited reduced activity against MCF-12A (IC_50_ = 188.20 ± 1.35μM) and MCF-7 cells (IC_50_ = 222. 60 ± 1.01) compared to dehydrocostus lactone (MCF-7: IC_50_ = 24.70 ± 1.25μM; MCF-12A: IC_50_ = 0.07 ± 0.07 μM), whilst 13-ethylenediamine derivative (**DHLC-4**) displayed improved cytotoxicity against MCF-7 cells (IC_50_ = 4.24 ± 1.29μM, SI = 34. 83) compared to the parent molecule (**DHLC**), with IC_50_ value of 24.70 ± 1.25 μM against the cell line. This suggests that addition of ethylene diamine at C-13 of dehydrocostus lactone improves anticancer activities on breast cancer cells compared to non-tumorigenic mammary epithelial cells (MCF-12A). In addition, primary amines derivatives including 13-dimethylamine (**DHLC-1)**, and 13-diethylamine (**DHLC-2)** showed improved cytotoxicity activities against HCC70 and MCF-7 cells compared to secondary amines derivatives as summarized in Table 2. The findings from the current study demonstrate the importance of synthetic modification of amino derivatives of dehydrocostus lactone having improved selectivity towards cancer cells over non-cancerous cells. This is a key step in identifying potential candidates for different breast cancer cells.

In related studies, synthesized 13-dimethylamino derivative of helenalin sesquiterpenes showed improved cytotoxicity activities (ED_50_ = 0.604 μg/mL) compared to helenalin (ED_50_ = 0.083 μg/mL) against Hep-2 (Human epidermoid carcinoma of the larynx) cells. Furthermore, dimethylamino adducts of costunolide displayed enhanced antiproliferative activities against leukemia cells (K562) with GI_50_ value of 4.4 μM compared to the parent molecule, costunolide (G1_50_ = 14.5 μM) [3, 19]. However, the derivative showed reduced antiproliferative activities against colon cancer cells (SW-620) with G1_50_ values of 9.1 μM compared to costunolide with G1_50_ value of 7.8 μM [3]. In addition, Srivastava et al. [20] reported the synthesis of costunolide derivatives. Among the derivatives, 3-methyl piperidine derivative displayed 2-fold greater cytotoxicity against colorectal cancer cells (SW-620) with IC_50_ value of 3.3 μM compared to costunolide (IC_50_ = 7.8 μM). Dehydrocostus lactone derivative having α-methylene-γ-lactone moiety was synthesized using Heck reactions by Ding et al. [21] and was evaluated for anticancer activities against doxorubicin resistant cell line (HL-60/A) cells. The results revealed that the derivative displayed inhibitory potency to HL-60/A with IC_50_ of 6.2 μM [21]. The derivative, however, presented no (IC_50_ > 50 μM) or reduced activity with IC_50_ values of between 31 and 52 μM against the cultured acute myelogenous leukemia cell line HL-60. It was therefore concluded that derivatives having substituents on the α-benzylidene-γ-lactone moiety can selectively inhibit doxorubicin-resistant cell line HL-60/A [21, 22].

### 6.1 ADME properties of the compounds

Since the biological activities of dehydrocostus lactone are attributed to α, *β*-methylene-γ-lactone, the addition of an amine into this moiety could of help to mask it from other nucleophiles and to increase the water solubility. Hence, the adsorption, distribution, metabolisms, and excretion properties (ADME) of the 13-amino derivatives of dehydrocostus lactone were further analyzed using SwissADME tools [23, 24]. This web server was selected because it is freely accessible and provides a robust and speedy computational method that can be used to estimate appraisal of the pharmacokinetics and toxicity of small molecules.

The physiochemical, pharmacokinetic properties, drug nature, and medicinal chemistry friendliness of the 13-amino derivatives were analyzed and results are summarized in Table 3. From the findings, all the synthetic compounds presented improved water solubility with an aqueous solubility descriptor (Log P (Ali)) ranging from lowest negative values of -2.92 to -3.82 compared to the parent dehydrocostus lactone (Table 3). The values were between the range of -3.99 and 0 in comparison to most of the standard drugs by the Food and Drug Administration (FDA) [24, 25]. In understating the water solubility properties of these derivatives, the ease of handling and formulation of potential therapeutic agents is improved and will ultimately facilitate future drug development. In addition, the compounds showed positive lipophilicity values (Log P (iLOGP) of between 2.41 and 3.42. The high positive values indicated high lipophilicity. However, the distribution of Log P (iLOGP) values is comparable to most values of between 0–10 for FDA drugs [25]. The lipophilicity of a compound is vital in any drug discovery efforts as it is related to its permeability through the biological membrane. The permeability could be decreased if the lipophilicity is too low whilst hydrophilic compounds are not able to diffuse through the membrane [24, 25]. Furthermore, the metabolisms prediction showed that the compounds inhibited the cytochromes CYPC19 but showed no inhibition of CYP2D6. However, all the compounds showed high gastrointestinal absorption, which suggested improved water solubility. In addition, all 13-amino derivatives demonstrated lead likeness properties with good synthetic accessibility scores compared to the parent molecule (dehydrocostus lactone) as illustrated in Table 3.

**Table 3:** The ADME properties of cytotoxic compounds.

Analysis	DHLC	1	2	3	4
**Water solubility**					
Log S (ESOL)	-2.92	-3.09	-3.32	-3.04	-2.24
Log S (Ali)	-2.82	-3.12	-3.24	-3.14	-2.32
**Physiochemical properties**					
No of heavy atoms	17	20	22	20	21
No of aromatic heavy atoms	0	0	0	0	0
No of rotatable bonds	3	2	4	3	4
No of H-bonds acceptors	2	3	3	3	4
No of H-bonds donors	0	1	0	1	2
Molar refractivity	67.74	80.63	90.34	80.63	83.34
Gastrointestinal absorption	High	High	High	High	High
CYPC19 inhibitor	Yes	No	No	No	No
CYP2D6 inhibitor	No	No	No	No	No
Log Kp (skin penetration) in cm/s	-5.84	-6.09	-6.05	-6.08	-6.04
**Drug-likeness**					
Bioavailability score	0.55	0.55	0.55	0.55	0.55
**Medicinal chemistry**					
Lead-likeness	No; Violation; MV < 250	Yes	Yes	Yes	Yes
Synthetic accessibility	3.84	4.13	4.31	4.09	4.15
**Lipophilicity**					
Implicit Log P (iLOGP)	2.59	2.95	3.42	3.14	2.41

**Fig 3:**
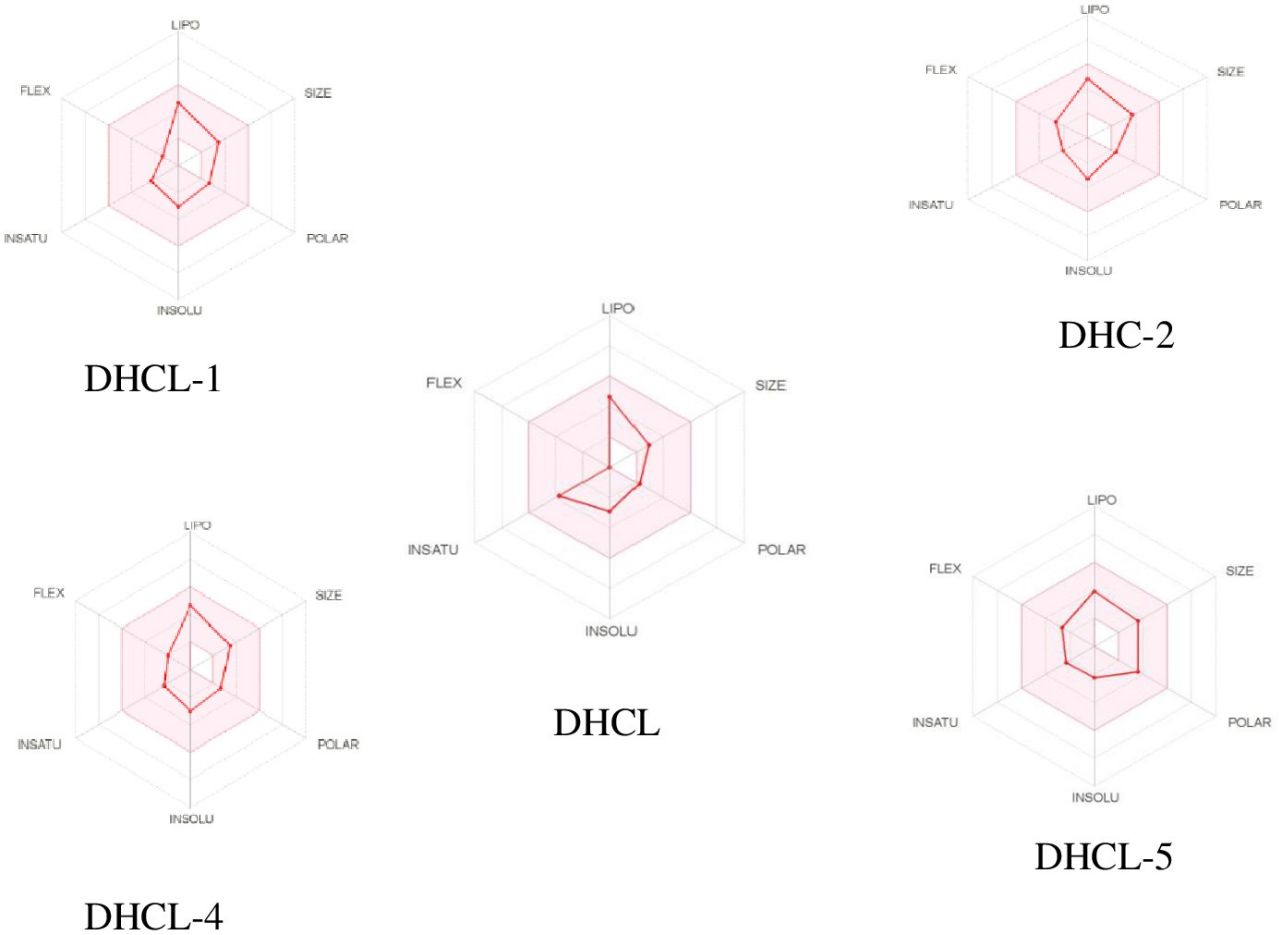
Bioavailability radar of the cytotoxic compounds. **Note**: The pink region indicates the optimal range for the properties predicted (size: Molecular weight between 140 and 400 g/mol, polarity: TPSA between 19 and 130 Å2, lipophilicity: XLOGP3 usually between −0.69 and + 4.9, saturation: fraction of carbons in the sp3 hybridization not less than 0.25, solubility: log *S* not higher than 6, and flexibility: no more than 9 rotatable bonds [24].

The bioavailability radar in Fig 3 indicated the optimal range for each property for most of the compounds [23]. Guaia-4(15),10(14)-dien-12-oic acid, 13-(ethylamino)-6-hydroxy-γ-lactone (**DHLC-3**) and 13-ethylenediamine dehydrocostus lactone (**DHLC-4**) showed the best bioavailability properties while dehydrocostus lactone (**DHLC**) showed poor flexibility properties [24].

### *In silico* molecular docking results

**Fig 4:**
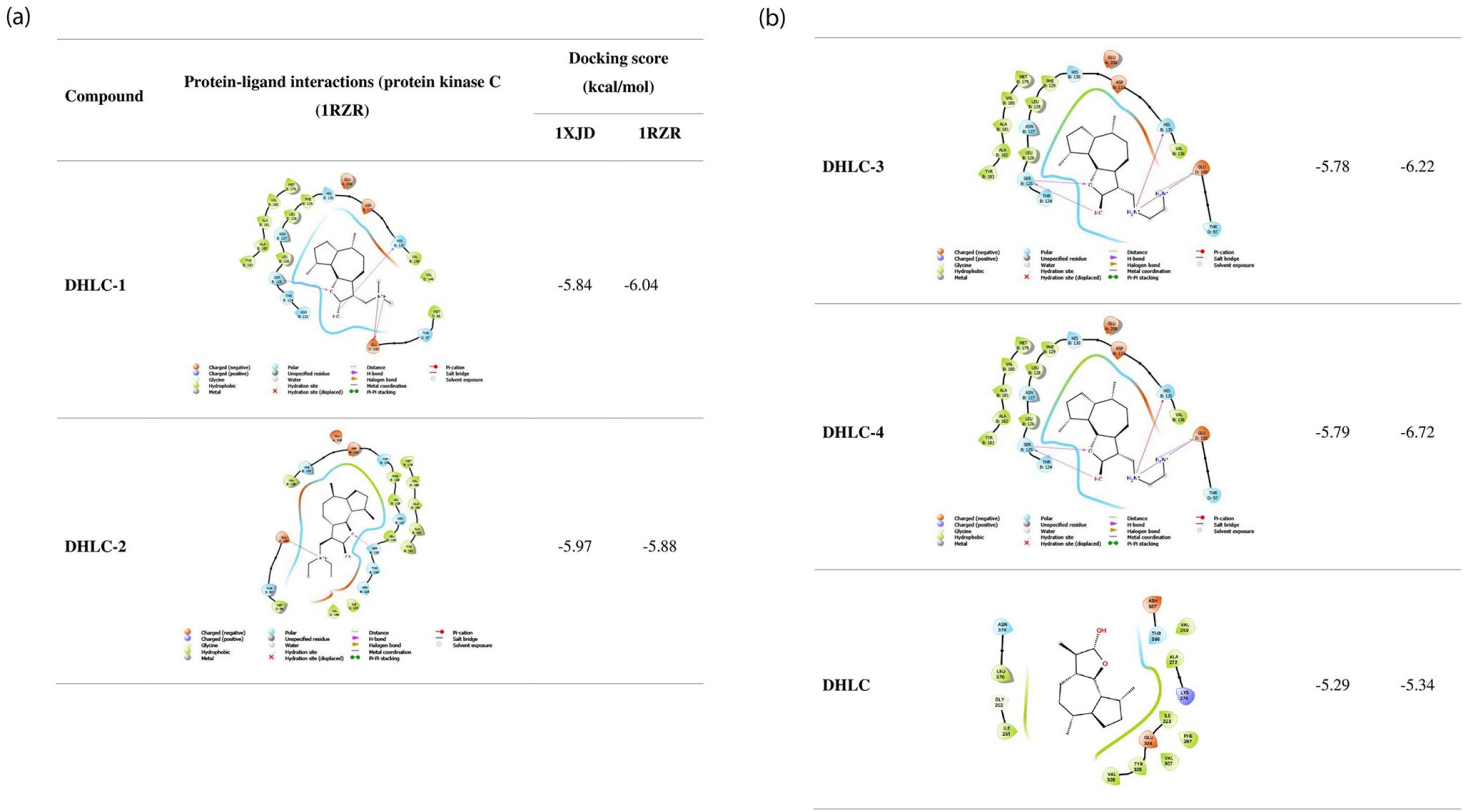
Molecular docking affinities of 13-amino derivatives of dehydrocostus lactone using protein kinase C (1RZR) and PKC theta (1XJD) enzymes.

Fig 4 shows the results of average docking scores, H-bond interactions, and other protein-ligand interactions for all synthetically modified compounds. Improved binding energies were recorded for 13-amino derivatives of dehydrocostus lactone for all the enzymes analyzed. 13-ethylenediamine dehydrocostus lactone (**DHLC-4**), and guaia-4(15),10(14)-dien-12-oic acid, 13-(ethylamino)-6-hydroxy-γ-lactone (**DHLC-3**) recorded the better binding energies of -7.33 and -5.97 kcal/mol, which were higher than the parent structure, dehydrocostus lactone (-5.34 kcal/mol) with the pivotal residues for PKC theta (1XJD) and -6.22 and -5.88 kcal/mol with the residues for 1ZRZ respectively.

**Fig 5:**
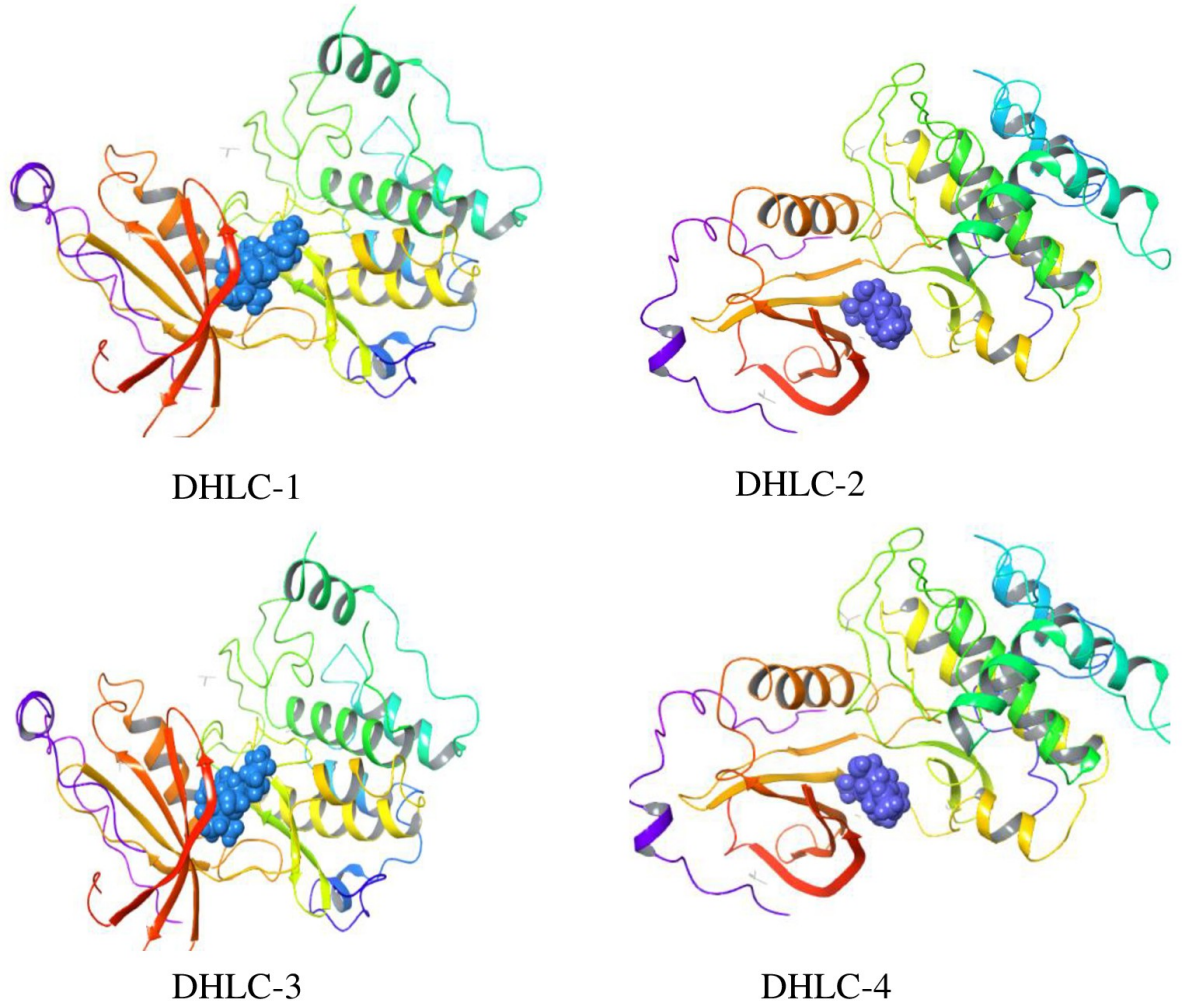
Best binding poses of 13-amino derivatives (DHLC-1-4) with 1ZRZ.

The high binding affinities of these derivatives were enhanced by the addition of amino groups on C-13, which were observed to form hydrogen bonds and pi interactions with the enzymes. Fig 5 shows the compounds (**DHLC-1-4)** posed in the active site pockets of the protein PKC theta (1XJD). The findings suggest that the activity of these compounds is associated with the ability of their skeletal structures to bind to specific enzymes.

## Conclusion

In summary, new 13-amino derivatives of dehydrocostus lactone (**1–4**) were synthesized through Michael’s addition reactions and were screened against breast cancer cell lines including hormone receptor-positive breast cancer (MCF-7), triple-negative breast cancer (HCC70), and non-tumorigenic mammary epithelial (MCF-12A) cell lines. The amino derivatives of dehydrocostus lactone, while displaying low micromolar toxicity overall to the breast cancer cells, also showed greatly improved selectivity for these breast cancer cells compared to the non-cancerous breast epithelial control cell line and were, in general more toxic to MCF-7 hormone receptor-positive breast cancer cells than the parent dehydrocostus lactone. The compounds further showed improved binding energies to PKC enzymes. Promising predicted ADME properties were also reported for all the compounds. Predicting the ADME properties of these derivatives is of importance in evaluating their drug-likeness, which could in turn be developed into potential drug candidates. The findings from the current study shows the promising potential of 13-amino derivatives of dehydrocostus lactone that can be investigated further to understand their structure-activity relationships about anticancer activities.

## Supporting information

S1 File (DOCX)

S2 File (ZIP)

S3 File (PNG)

## References

[1] Santana A, Molinillo JMG, Macías FA. Trends in the synthesis and functionalization of guaianolides. Eur. J. Org. Chem. 2015; (10):2093–2110. doi: 10.1002/ejoc.201403244

[2] Drew DP, Krichau N, Reichwald K, Simonsen HT. Guaianolides in Apiaceae: Perspectives on pharmacology and biosynthesis. Phytochem Rev. 2009; 8(3):581–599. doi: 10.1007/s11101-009-9130-z

[3] Woods JR, Mo H, Bieberich AA, Alavanja T, Colby DA. Amino derivatives of the sesquiterpene lactone class of natural products as prodrugs. Med.Chem.Comm. 2013; 4(1): 27–33. doi: 10.1039/C2MD20172K

[4] Dewick MP. Medicinal Natural Products: a biosynthetic approach. 3^nd^ edition. John Wiley & Sons, United Kingdom. 2002; 203–211.

[5] Choi JH, Ha J, Park JH, Lee JY, Lee YS, Park HJ, et al. Costunolide triggers apoptosis in human leukemia U937 cells by depleting intracellular thiols. Jpn J. Cancer Res. 2002; 93:1327–1333. doi: 10.1111/j.1349-7006.2002.tb01241.x 12495472 PMC5926928

[6] Lai HC, Singh NP, Sasaki T. Development of artemisinin compounds for cancer treatment. *Invest*. *New Drugs*. 2013; 31:230–246. doi: 10.1007/s10637-012-9873-z 22935909

[7] Pyun H, Kang U, Seo EK, Lee K. Dehydrocostus lactone, a sesquiterpene from *Saussurea lappa Clarke*, suppresses allergic airway inflammation by binding to dimerized translationally controlled tumour protein. Phytomedicine. 2018; 43:46–54. doi: 10.1016/j.phymed.2018.03.045 29747753

[8] Singireesu SSNR, Mondal SK, Misra S, Yerramsetty SKSB. Dehydrocostus lactone induces prominent apoptosis in kidney distal tubular epithelial cells and interstitial fibroblasts along with cell cycle arrest in ovarian epithelial cells. Biomed. Pharmacother. 2018; 99:956–969. doi: 10.1016/j.biopha.2018.01.099 29710496

[9] Tabata K, Nishimura Y, Takeda T, Kurita M, Uchiyama T, Suzuki T. Sesquiterpene lactones derived from *Saussurea lappa* induce apoptosis and inhibit invasion and migration in neuroblastoma cells. J. Pharmacol. Sci. 2015; 127(4):397–403. doi: 10.1016/j.jphs.2015.01.002 25953266

[10] Kushwaha N, Saini RK, Kushwaha SKS. Synthesis of some amide derivatives and their biological activity. Int. J. Chem. Technol. Res. 2011; 3(1):203–209.

[11] Jeffrey T, Kuethe I, Davies W. Preparation of 2-arylindole-4-carboxylic amide derivatives. Tetrahedron. 2006; 62:11381–11390. doi: 10.1016/j.tet.2006.05.007

[12] De la mare J, Lawson JC, Chiwakata MT, Denzil RB, Andriena LE, Gregory LB. Quinones and halogenated monoterpenes of algal origin show anti-proliferative effects against breast cancer cells *in vitro*. Investigational New Drugs. 2012; 30:2187–2200. doi: 10.1007/s10637-011-9788-0 22249429

[13] Mbaba M, de la Mare J-A, Sterrenberg JN, Kajewole D, Maharaj S, Edkins AL, et al. Novobiocin–ferrocene conjugates possessing anticancer and antiplasmodial activity independent of HSP90 inhibition, J. Biol. Inorg. Chem. 2019; 24:139–149. doi: 10.1007/s00775-018-1634-9 30542925

[14] Bernstein FC, Koetzle TF, Williams GJ, Meyer EF, Brice MD, Rodgers JR, et al. The Protein D a t a Bank: A computer based archival file for macromolecular structures. 1977; 535–42. doi: 10.1016/s0022-2836(77)80200-3 875032

[15] Biovia DS. BIOVIA Workbook. San Diego: 2016. Dassault Systemes.

[16] Tiina L, Sami H, Kristiina W. Synthesis and applications of secondary amine derivatives of (+)-dehydroabietylamine in chiral molecular recognition. Org. Biomol. Chem. 2015; 13:10548–10555. doi: 10.1039/c5ob01667c 26337032

[17] Danil P, Zarezin V, Khrustalev N, Valentine G. Diastereoselectivity of Azido-Ugi reaction with secondary amines: stereo-selective synthesis of tetrazole derivatives. J. Org. Chem. 2017; 82:6100–6107. doi: 10.1021/acs.joc.7b00611 28558241

[18] Hisashi M, Tadashi K, Yasunao I, Toshio M, Masayuki Y. Absolute stereo structures and syntheses of saussureamines A, B, C, D, and E, amino acid sesquiterpene conjugates with gastroprotective effect, from the roots of *Saussurea lappa*. Tetrahedron. 2000; 56:7763–7777. doi: 10.1016/S0040-4020(00)00696-7

[19] Unde NR, Hiremath SV, Kulkarni GH, Kelkar GR. Amino derivatives of sesquiterpenes lactone class of natural products prodrugs. Tetrahedron Lett. 1968; 9:4861–4862.

[20] Srivastava SK, Abraham A, Bhat B, Jaggi M, Singh AT, Sanna VKG, et al. Synthesis of 13-amino costunolide derivatives as anticancer agents. Bioorg. Med. Chem. Lett. 2006; 16:4195–4199. doi: 10.1016/j.bmcl.2006.05.083 16766184

[21] Ding YH, Fan HX, Long J, Zhang Q, Chen Y. The application of Heck reaction in the synthesis of guaianolide sesquiterpene lactones derivatives selectively inhibiting resistant acute leukemic cells. Bioorg. Med. Chem. Lett. 2013; 23:6087–6092. doi: 10.1016/j.bmcl.2013.09.028 24095093

[22] Robinson A, Kumar TV, Sreedhar E, Naidu VG, Krishna SR, Babu KS, et al. A new sesquiterpene lactone from the roots of Saussurea lappa: structure-anticancer activity study. Bioorg. Med. Chem. Lett. 2008; 18:4015–4017. doi: 10.1016/j.bmcl.2008.06.008 18579374

[23] Jia C.-Y, Li J.-Y, Hao G.-F, Yang G.-F. A drug-likeness toolbox facilitates ADMET study in drug discovery. Drug Discovery Today. 2020; 25:248–258. doi: 10.1016/j.drudis.2019.10.014 31705979

[24] Daina A, Michielin O, Zoete V. SwissADME: a free web tool to evaluate pharmacokinetics, drug-likeness and medicinal chemistry friendliness of small molecules. Sci. Rep. 2017; 7:42717–42721. doi: 10.1038/srep42717 28256516 PMC5335600

[25] Durán-Iturbide AN, Bárbara ID, Medina-Franco JL. *In Silico* ADME/Tox Profiling of Natural Products: A Focus on BIOFACQUIM. ACS Omega. 2020; 5:16076–16084. doi: 10.1021/acsomega.0c01581 32656429 PMC7346235

