# Supplementary material for: 13-amino derivatives of dehydrocostus lactone display greatly enhanced selective toxicity against breast cancer cells and improved binding energies to protein kinases in silico
Source: PLoS One. 2022 Aug 23;17(8):e0271389. doi: 10.1371/journal.pone.0271389 (PMC9397875; doi:10.1371/journal.pone.0271389)
Supplement: S1 File — (DOCX) [file pone.0271389.s001.docx]

**13-amino derivatives of dehydrocostus lactone display greatly enhanced selective toxicity against breast cancer cells and improved binding energies to protein kinases *in silico***

Douglas Kemboi ^1, 2*^, Moses K. Langat ^3^, Xavier Siwe-Noundou ^4^, Tendamudzimu Tshiwawa^2^ Rui W.M. Krause ^2^, Candace Davison,^5^, Christie Jane Smit^5^, Jo-Anne de la Mare ^5^, Vuyelwa Jacqueline Tembu ^1^**^*^**

^1^ Department of Chemistry, Tshwane University of Technology, Pretoria, 0001, South Africa.

^2^ Department of Chemistry, Rhodes University, Makhanda (Grahamstown), 6140, South Africa.

^3^ Royal Botanic Gardens Kew, Kew Green, Richmond, Surrey, TW9 3AE, UK.

^4^ Pharmaceutical Sciences Department, Sefako Makgatho Health Sciences University, Pretoria, 0204, South Africa.

^5^ Department of Biochemistry and Microbiology, Rhodes University, Makhanda (Grahamstown), 6140, South Africa.

^*^ **Corresponding authors**: [kemboidouglas01@gmail.com](mailto:kemboidouglas01@gmail.com) (D.K); [TembuVJ@tut.ac.za](mailto:TembuVJ@tut.ac.za) (J.T); Tel.: +27-12-382-6288 (J.T).

**Abstract**

The biological activities of dehydrocostus lactone and its analogues are suggested to be mediated by the lactone ring and *α,β*-methylene-*γ*-lactone. However, few studies exist on the structure-activity relationship of 13-amino derivatives of dehydrocostus latone. In this study new 13-amino derivatives of dehydrocostus lactone **DHLC** (**1-4**) were synthesized through Michael addition reactions, and were screened against three different breast cancer cell lines, namely hormone receptor positive breast cancer (MCF-7), triple-negative breast cancer (HCC70), and non-tumorigenic mammary epithelial (MCF-12A) cell lines. Dehydrocostus lactone (**DHLC**) exhibited IC_50_ values of 1.11 (selectivity index (SI) = 0.06), 24.70 (SI = 0.01) and 0.07 µM against HCC70, MCF-7 and MCF-12A cells, respectively. All the amino derivatives, except **DHLC-3** displayed low micromolar IC_50_ values (ranging from 0.07- 4.24 µM) against both breast cancer cell lines, with reduced toxicity towards MCF-12A non-tumorigenic mammary epithelial cells (SI values ranging from 6.00 - 126.86). **DHLC-1** and D**HLC-2** demonstrated the greatest selectivity for the MCF-7 cells (with SI of 121 and 126.86 respectively) over the MCF-12A cells. This reveals that, overall, the derivatives display greatly improved selectivity for breast cancer over non-tumorigenic mammary epithelial cells, with between 100-fold and 12 000-fold higher SI values. The improved docking scores were recorded for all the 13-amino dehydrocostus lactone derivatives for the enzymes analyzed. Compounds **DHLC-4**, and **DHLC-3** recorded higher docking scores of -7.33 and -5.97 Kca/mol respectively, compared to the parent structure, dehydrocostus lactone (-5.34 Kca/mol) for protein kinase (PKC) theta (1XJD) and -6.22 and -5.88 Kca/mol, respectively for protein kinase iota (1RZR). The compounds further showed promising predicted adsorption, distribution, metabolisms and excretion (ADME) properties. Predicting the ADME properties of these derivatives is of importance in evaluating their drug-likeness, which could in turn be developed into potential drug candidates.

**Key words**: Dehydrocostus lactone, synthesis, breast cancer, MCF-7, HCC70, MCF-12A, ADME properties, molecular docking.

**Table S1**: The ^1^H (400 MHz) and ^13^C NMR (100.6 MHz) data of synthesized compounds (**1-4**)

|  | DHLC -1 | | DHLC -2 | | DHLC -3 | | DHLC -4 | |
| --- | --- | --- | --- | --- | --- | --- | --- | --- |
| **No** | **^13^C (δ_c_)** | ***δ*_H_ (*J* in Hz)** | **^13^C (δ_c_)** | ***δ*_H_ (*J* in Hz)** | **^13^C (δ_c_)** | ***δ*_H_ (*J* in Hz)** | **^13^C (δ_c_)** | ***δ*_H_ (*J* in Hz)** |
| 1 | 47.1 (CH) | 2.72 (1H, *m*) | 47.1 (CH) | 2.78 (1H, *m*) | 47.8 (CH) | 2.91 (1H, *m*) | 47.5 (CH) | 2.37 (1H, *m*) |
| 2*α* | 32.6 (CH_2_) | 1.98 (1H, *m*) | 32.6 (CH_2_) | 2.40 (1H, *m*) | 32.5 (CH_2_) | 1.39 (1H, *m*) | 32.5 (CH_2_) | 2.54 (1H, *m*) |
| 2*β* |  | 2.01 (1H, *m*) |  | 2.27 (1H, *m*) |  | 2.16 (1H, *m*) |  | 2.13 (1H, *m*) |
| 3*α* | 30.2 (CH_2_) | 3.86 (1H, *t, J*=2.1) | 30.2 (CH_2_) | 3.82 (1H, *m*)  - | 30.2 (CH_2_) | 2.54 (1H, *t, J* = 2.1) | 30.2 (CH_2_) | 1.97 (1H, *m*) |
| 3*β* |  | - |  |  |  | 1.97(1H, *m*) |  | 1.85 (1H, *m*) |
| 4 | 151.9 (C) | - | 151.8 (C) | - | 151.8 (C) | - | 151.9 (C) | - |
| 5 | 52.0 (CH) | 2.83 (1H, *m*) | 52.1 (CH) | 2.51 (1H, *m*) | 51.9 (CH) | 2.85 (1H, *m*) | 51.9 (CH) | 2.83 (1H, *m*) |
| 6 | 85.4 (CH) | 2.75 (1H, *t*, *J* = 6.4) | 85.4 (CH) | 2.71 (1H, *m*) | 85.8 (CH) | 3.98 (1H, *t*, *J* = 6.0) | 85.4 (CH) | 3.96 (1H, *t*, *J* = 6.0) |
| 7 | 45.5 (CH) | 2.63 (1H, *m*) | 45.9 (CH) | 2.39 (1H, *m*) | 45.4 (CH) | 2.25 (1H, *m*) | 45.0 (CH) | 2.42 (1H, *m*) |
| 8*α* | 32.9 (CH_2_) | 2.39 (1H, *m*) | 33.0 (CH_2_) | 3.87 (1H, *m*) | 32.7 (CH_2_) | 2.16 (1H, *m*) | 32.7 (CH_2_) | 2.50 (1H, *m*) |
| 8*β* |  | 1.84 (1H, *m*) |  | - |  | 1.39 (1H, *m*) |  | 2.12 (1H, *m*) |
| 9*α* | 37.8 (CH_2_) | 2.51 (1H, *m*) | 37.7 (CH_2_) | 2.15 (1H, *m*) | 37.6 (CH_2_) | 2.51 (1H, *m*) | 37.7 (CH_2_) | 2.07 (1H, *m*) |
| 9*β* |  | 2.17 (1H, *m*) |  | 1.77 (1H, *m*) |  | 2.06 (1H, *m*) |  | 2.49 (1H, *m*) |
| 10 | 150.6 (C) | - | 150.2 (C) | - | 149.7 (C) | - | 150.7 (C) | - |
| 11 | 46.9 (CH) | 2.73 (1H, *t, J* = 1.9) | 45.9 (CH) | 2.77 (1H, *m*) | 47.8 (CH) | 2.73 (1H, *t, J* = 1.9) | 49.4 (CH) | 2.73 (1H, *t, J* = 1.9) |
| 12 | 177.7 (C) | - | 177.9 (C) | - | 178.0 (C) | - | 177.8 (C) | - |
| 13*α* | 58.8 (CH_2_) | 2.64 (1H, *d, J* = 1.2) | 52.9 (CH_2_) | 2.48 (1H, *m*) | 47.8 (CH_2_) | 2.91 (1H, *d, J* = 1.2) | 47.7 (CH_2_) | 2.88 (1H, *m*) |
| 13*β* |  | 2.51 (1H, *d, J* = 1.2) |  | 2.38 (1H, *m*) |  | 2.45 (1H, *d, J* = 1.2) |  | 2.35 (1H, *m*) |
| 14*α* | 109.2 (CH_2_) | 4.79 (1H, *brs*) | 109.1 (CH_2_) | 4.79 (1H, *brs*) | 109.2 (CH_2_) | 5.07 (1H, *brs*) | 109.2 (CH_2_) | 5.06 (1H, *brs*) |
| 14*β* |  | 5.12 (1H, *brs*) |  | 4.68 (1H, *brs*) |  | 5.20 (1H, *brs*) |  | 5.20 (1H, *brs*) |
| 15 | 111.7 (CH_2_) | 4.96 (1H, *brs*) | 111.6 (CH_2_) | 5.12 (1H, *brs*)  4.97 (1H, *brs*) | 111.9 (CH_2_) | 4.90 (1H, *brs*) | 112.0 (CH_2_) | 4.89 (1H, *brs*) |
|  |  | 4.90 (1H, *brs*) |  |  |  | 4.80 (1H, *brs*) |  | 4.79 (1H, *brs*) |
| 16 | 45.9 (CH_3_) | 2.17 (3H. *s*) | 47.1 (CH_2_) | 2.41 (1H, *m*) | 44.3 (CH_2_) | 1.37 (1H, *m*) | 52.7 (CH_2_) | 2.71(1H, *m*) |
| 17 | 45.9 (CH) | 2.17 (1H, *s*) |  | 2.34 (1H, *m*) | 14.9 (CH_3_) | 1.16 (3H, *t, J = 8*) | 41.6 (CH_2_) | 2.82 (1H, *m*) |
| 18 | - | - | 11.7 (CH_3_) | 0.92 (3H, *t, J* = 19.4) | - | - | - | - |
| 19 | - | - | 11.7 (CH_3_) | 0.92 (3H, *t, J* = 19.4) | - | - | - | - |

Appendix A1: ^1^H NMR spectrum of dehydrocostus lactone (**DHLC**) in CDCl_3_

Appendix A2: ^13^C NMR spectrum of DHLC in CDCl_3_

Appendix A3: ^1^H-^1^H COSY spectrum of DHLC in CDCl_3_

Appendix A4: HSQC spectrum of DHLC in CDCl_3_

Appendix A5: HMBC spectrum of DHLC in CDCl_3_

Appendix A6: NOESY spectrum of DHLC in CDCl_3_

Appendix A7: MS spectrum of DHLC

**Appendix A8**: IR spectrum of **DHLC**


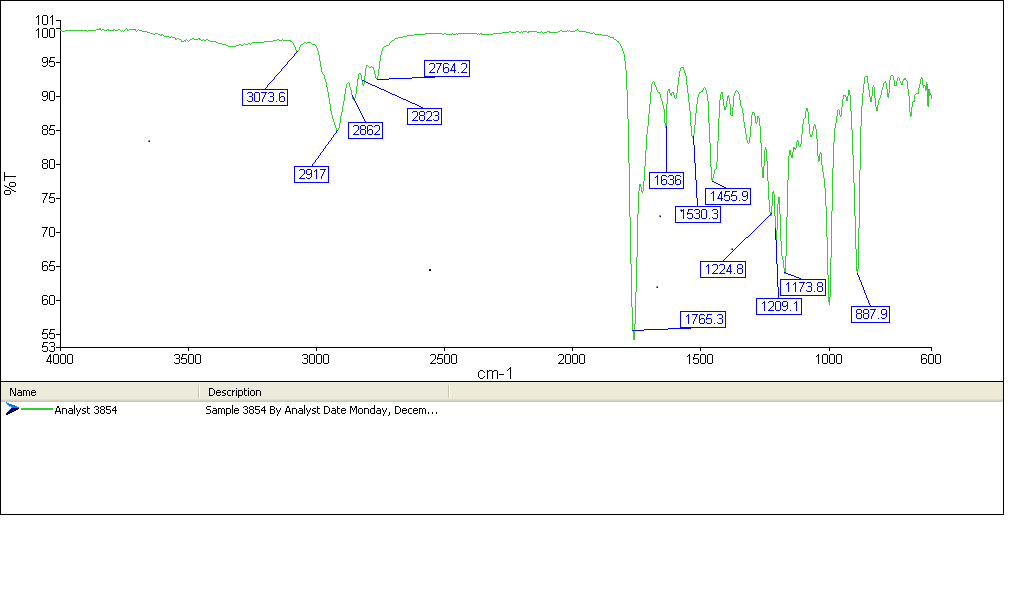


Appendix A9: ^1^H NMR spectrum of DHLC-1 in CDCl_3_

O

O

N

1

2

3

4

6

7

8

9

10

11

12

13

14

15

16

17

H

H

H

H

H

Appendix A10: ^13^C NMR spectrum of DHLC-1 in CDCl_3_

O

O

N

1

2

3

4

6

7

8

9

10

11

12

13

14

15

16

17

H

H

H

H

H

Appendix A11: ^1^H- ^1^H COSY spectrum of DHLC-1 in CDCl_3_

Appendix A12: HSQC spectrum of DHLC-1 in CDCl_3_

Appendix A13: HMBC spectrum of DHLC-1 in CDCl_3_

Appendix A14: NOESY spectrum of DHLC-1 in CDCl_3_

Appendix A15: DEPT spectrum of DHLC-1 in CDCl_3_

**Appendix A16**: IR spectrum of **DHLC-1**

Appendix A17: MS spectrum of DHLC-1

Appendix A18: ^1^H NMR spectrum of DHLC-2 in CDCl_3_

O

O

N

1

2

3

4

6

7

8

9

10

11

12

13

14

15

16

17

18

19

H

H

H

H

H

Appendix A19: ^13^C NMR spectrum of DHLC-2 in CDCl_3_

O

O

N

1

2

3

4

6

7

8

9

10

11

12

13

14

15

16

17

18

19

H

H

H

H

H

Appendix A20: ^1^H-^1^H COSY spectrum of DHLC-2 in CDCl_3_

Appendix A21: HSQC spectrum of DHLC-2 in CDCl_3_

Appendix A22: HMBC spectrum of DHLC-2 in CDCl_3_

Appendix A23: DEPT spectrum of DHLC-2 in CDCl_3_

Appendix A24: NOESY spectrum of DHLC-2 in CDCl_3_

Appendix A25: MS spectrum of DHLC-2 Appendix A26: IR spectrum of DHLC-2

Appendix A27: ^1^H NMR spectrum of DHLC-3 in CDCl_3_

O

O

H

N

1

2

3

4

6

7

8

9

10

11

12

13

14

15

16

17

H

H

H

H

H

Appendix A28: ^13^C NMR spectrum of DHLC-3 in CDCl_3_

O

O

H

N

1

2

3

4

6

7

8

9

10

11

12

13

14

15

16

17

H

H

H

H

H

Appendix A29: ^1^H-^1^H COSY spectrum of DHLC-3 in CDCl_3_

Appendix A30: HSQC spectrum of DHLC-3 in CDCl_3_

Appendix A31: HMBC spectrum of DHLC-3 in CDCl_3_

Appendix A32: DEPT spectrum of DHLC-3 in CDCl_3_

Appendix A33: NOESY spectrum of DHLC-3 in CDCl_3_

Appendix A34: MS spectrum of DHLC-3

**Appendix A35**: IR spectrum of **DHLC-3**

Appendix A36: ^1^H NMR spectrum of DHLC-4 in CDCl_3_

O

O

H

N

1

2

3

4

6

7

8

9

10

11

12

13

14

15

16

17

H

H

H

H

H

NH

2

Appendix A37: ^13^C NMR spectrum of DHLC-4 in CDCl_3_

O

O

H

N

1

2

3

4

6

7

8

9

10

11

12

13

14

15

16

17

H

H

H

H

H

NH

2

Appendix A38: ^1^H-^1^H COSY spectrum of DHLC-4 in CDCl_3_

Appendix A39: HSQC spectrum of DHLC-4 in CDCl_3_

Appendix A40: HMBC spectrum of DHLC-4 in CDCl_3_

Appendix A41: DEPT spectrum of DHLC-4 in CDCl_3_

Appendix A42: NOESY spectrum of DHLC-4 in CDCl_3_

Appendix A43: MS spectrum of DHLC-4

**Appendix A44**: IR spectrum of **DHLC-4**
