# Supplementary figures and images for: 13-amino derivatives of dehydrocostus lactone display greatly enhanced selective toxicity against breast cancer cells and improved binding energies to protein kinases in silico
Source: PLoS One. 2022 Aug 23;17(8):e0271389. doi: 10.1371/journal.pone.0271389 (PMC9397875; doi:10.1371/journal.pone.0271389)

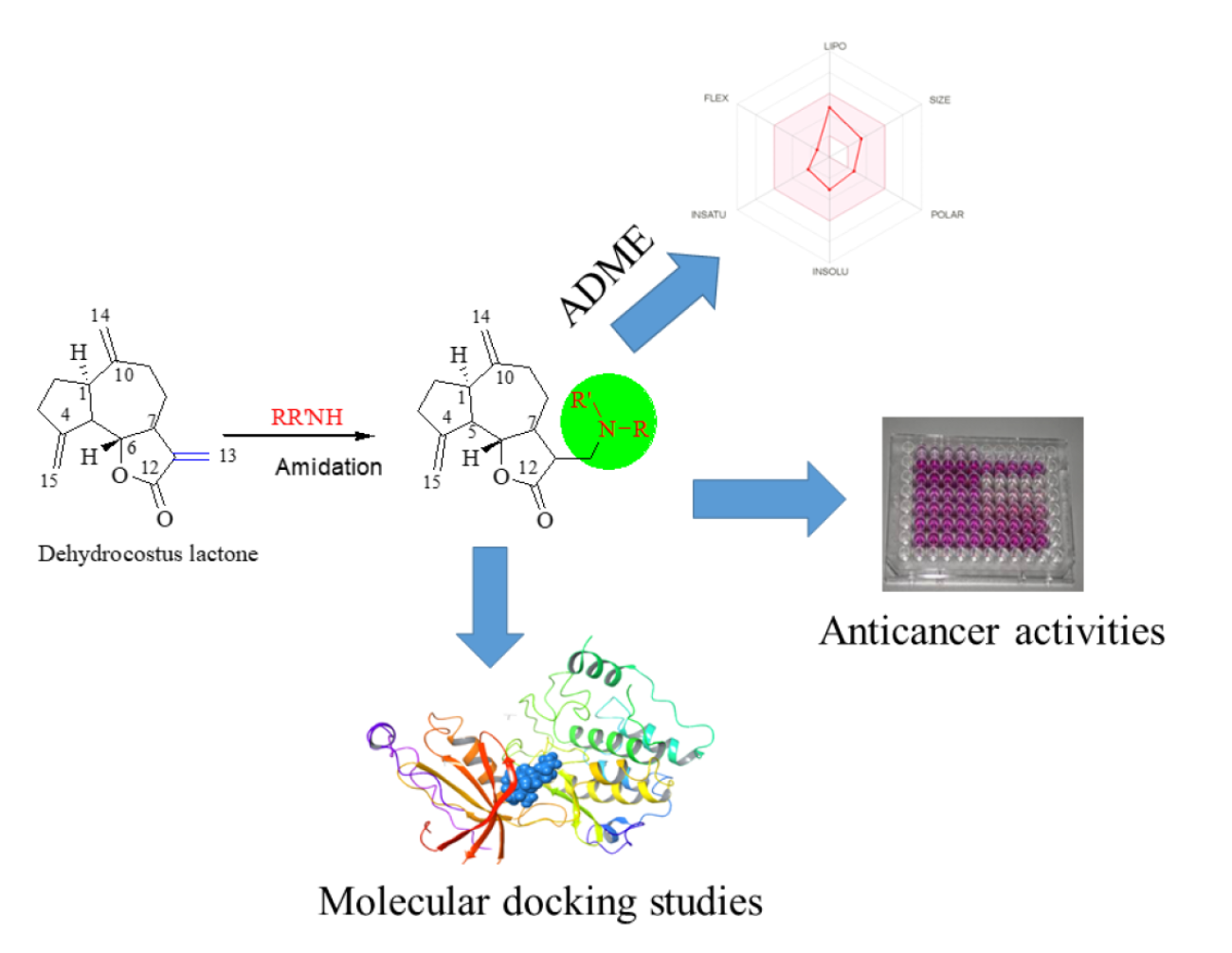

Supplement: S3 File — (PNG) [file pone.0271389.s003.png]
